# Numerical Investigation of Degradation of 316L Steel Caused by Cavitation

**DOI:** 10.3390/ma14113131

**Published:** 2021-06-07

**Authors:** Artur Maurin

**Affiliations:** Institute of Fluid-Flow Machinery, Polish Academy of Sciences, Hydropower Department, Fiszera 14 St., 80-231 Gdańsk, Poland; amaurin@imp.gda.pl; Tel.: +48-58-5225-186

**Keywords:** cavitation erosion, micro-jet, numerical simulation, 316L steel

## Abstract

The degradation process of 316L stainless steel caused by cavitation was investigated by means of finite element analysis. The damage characteristics of metal specimens subjected to the cavitation bubble collapse process were recreated by simulation with a micro-jet water hammer. The simulation results were compared with the cavitation pits created in the experimental tests. In the experiment, different inlet and outlet pressures in a test chamber with a system of barricade exciters differentiated the erosion process results. Hydrodynamic cavitation caused uneven distribution of the erosion over the specimens’ surface, which has been validated by roughness measurements, enabling localisation and identification of the shape and topography of the impact pits. The erosion rate of the steel specimens was high at the beginning of the test and decreased over time, indicating the phase transformation and/or the strain-hardening of the surface layer. A numerical simulation showed that the impact of the water micro-jet with a velocity of 100 m/s exceeds the tensile strength of 316L steel, and produces an impact pit. The subsequent micro-jet impact on the same zone deepens the pit depth only to a certain extent due to elastoplastic surface hardening. The correlation between post-impact pit geometry and impact velocity was investigated.

## 1. Introduction

Today, the hydropower machinery is often forced to work beyond the best efficiency point conditions in order to adjust to energy market requirements. On the other hand, there is a tendency to increase the specific speed of newly built machines. Both conditions lead the cavitation erosion effects to be noticed more often.

Cavitation is a multiphase phenomenon, involving multiple vapour bubbles (gas nuclei) to be created and collapse, that normally occurs at high frequency in a turbulent flow, during rapid acceleration of the liquid. This causes the pressure waves to generate and propagate through the liquid and structure. Further, these pressure waves can damage the walls of channels or blades (in the case of hydraulic machinery), as in the erosion process. To counter adverse erosion effects on material structures, a good understanding of degradation mechanisms is necessary.

Collapsing cavitation bubble liquid micro-jets together with the shockwaves radiated upon collapse (nucleation process) appear to play the most essential roles in the degeneration of solids submerged in cavitating liquids. The bubble collapse that occurs nearby a solid boundary always causes the micro-jet to be directed towards the solid boundary, as the radial flow of liquid is restricted by the solid boundary in proximity of the bubble wall. In consequence, the liquid pressure at the bubble wall close to the solid surface is at minimum, while the pressure at the bubble wall furthermost from the solid surface is at maximum. Fundamental physical processes of bubble dynamics and the phenomenon of cavitation itself are well established. The nucleation process is described in both the theory and observations for flowing and quiescent liquids [1].

From the structural mechanics point of view, the permanent deformation or pit is formed when the local equivalent stresses exceed the material yield stress, resulting in the initiation of slip and dislocation movements, twinning and/or phase transformations, and growth cracks in metals [2]. On the microscopic level, as plastic deformation occurs (micro-yielding), atomic planes slip past each other through the movement of dislocations. These atomic-scale deformations release energy in the form of elastic waves (acoustic emissions) which are traveling through the structure [2]. Generally, the amount of energy released by the micro-jet impact and the amplitude of the waveform are related to the magnitude and velocity of the source event.

The theoretical upper limit of micro-jet impact velocity was specified to 1202.9 m/s in case of the collapse of a 3.5 mm-radius cavitation bubble, as assessed by Xie [3]. However, the energy of the micro-jet dissipated in the fluid significantly reduces this value. Determining the actual velocity of micro-jets has been the subject of many studies [4,5,6,7,8]. The impact velocity can range from 20 m/s to 500 m/s or even more. However, the velocity value depends on the size of the cavitation bubble, the water vapour content, and the thickness of the water boundary layer. Lauterborn and Bolle [9] found in their investigation that the maximum velocity of a micro-jet is 120 m/s. Dular et al. [6] obtained the maximum micro-jet velocity in the order of 175 m/s. Similar results (the water jet velocity in the range from 179 to 232 m/s) were obtained by Wright et al. [10]. In contrast, Field et al. [11], Bourne [12], and Smith and Kinslow [13] showed that the micro-jet speed can be in the range 400–600 m/s. Therefore, for simulations presented in this paper, the velocity of the micro-jet was chosen in range from 100 m/s to 500 m/s. As for the impact magnitude, according to References [14,15,16], the micro-jet causes the maximal damage to the solid surfaces at an angle of 90 degrees. 

In this paper, the analysis of 316L stainless steel degradation was performed based on finite element (FE) structural strength calculations of two subsequent impacts of liquid micro-jets at high velocities. The detailed overview of this approach is presented in Section 2. The correlation between post-impact pit geometry and impact velocity was investigated (Section 3) in order to provide a better understanding of the relationship between changes in surface deformation and material hardness. The influence of micro-jet impact on stress distribution in the surface layer of 316L stainless steel is discussed in Section 4.

## 2. Materials and Methods

The 316L stainless steel material is commonly used in hydropower machinery. Therefore, it is often subjected to cavitation type damage. The 316L steel that was used in the experimental tests have a chemical composition designated according to the EN 10204 3.1B specification, presented in Table 1.

The stress response characteristic of the micro-jet hammering process was explored by a comparison of the impact pit depths created at different impact velocities and pit geometries created in experimental tests [17]. In [17], the test samples were made from X8CrNiTi18-10 steel which is equivalent to 316L steel.

**Figure 1:**
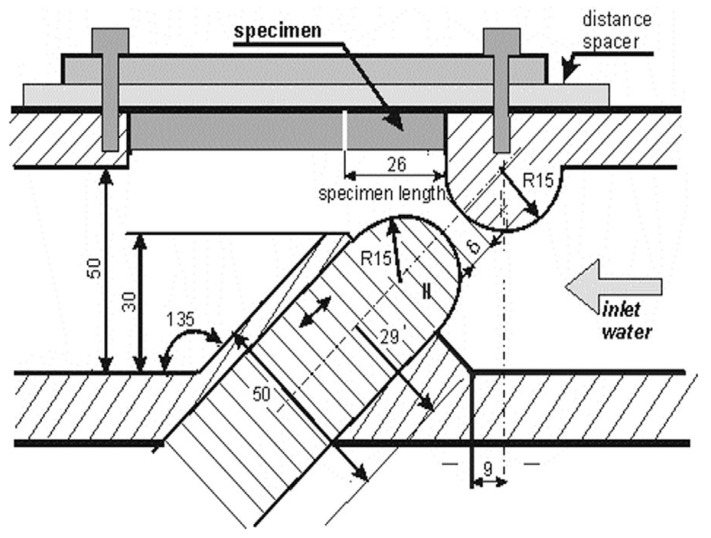
Schematic of the cavitation chamber test device [17] (Reprinted from “Effect of cavitation intensity on degradation of X6CrNiTi18-10 stainless steel” by A.K. Krella, A. Krupa, Wear, Volumes 408–409, 2018, Pages 180-189, ISSN 0043-1648, Copyright under license no. 5057500490519 (2021), with permission from Elsevier).

The referenced experimental tests were performed using a cavitation chamber with a system of barricade exciters. The schematic diagram of the cavitation chamber shown in Figure 1 was described in Reference [2]. The cavities (cavitating vortices and bubbles) are generated in the zone behind the upper barricade exciter due to the sudden pressure drop, which is produced by a change in flow velocity along the slot between two semi-cylindrical barricades.

The test device allowed to perform erosion experiments at various cavitation conditions, which were adjusted by the inlet and outlet pressures, the slot width, and the spacer thickness. The pressures were regulated by the inlet and outlet valves. The inlet and outlet valves were located approximately 1 m and 5 m from the from the test chamber, respectively. The specimen was located next to the upper barricade, as shown in Figure 1. In the referenced experiment [17], the cavitation tests were carried out at the inlet pressures p1 = 600, 700, 800, and 900 kPa, and the outlet pressures were an effect of a completely open outlet valve and were equalled to p2 = 123, 125, 129, and 132 kPa, respectively. The slot width was equal to δ = 5.5 mm, and the distance spacer of 4 mm thickness was used. 

The difference in inlet and outlet pressures was proportional to the cavitation erosion intensity. The total cavitation test duration was 600 min. After the cavitation tests, microscopic observations of the eroded surface of the specimens tested at p1 = 600 kPa and 900 kPa were performed using a scanning electron microscope EVO-40 (Zeiss, Oberkochen, Germany). The arithmetic mean of surface roughness measurement values (micro-jet impact pit depths) were calculated from the experimental data (presented in Section 3).

**Figure 2:**
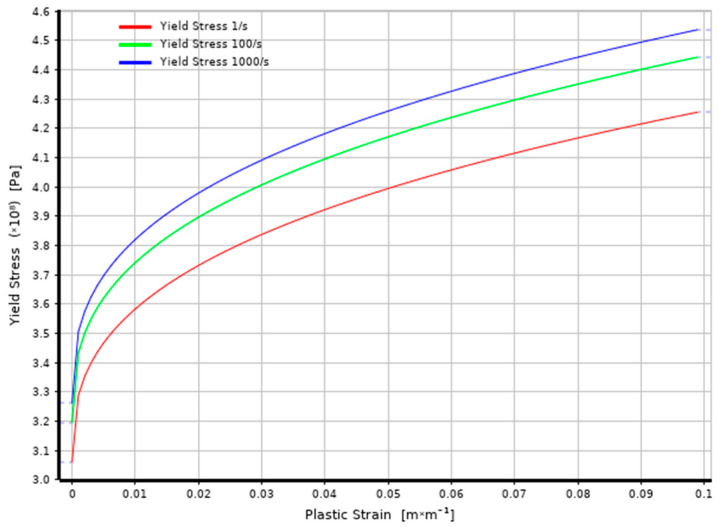
Johnson–Cook strength characteristics for 316L stainless steel.

The material properties adopted for the 316L stainless steel micro-jet hammering simulation are presented in Table 2. The Johnson and Cook [18] model was employed in the simulation with strength characteristics presented in Figure 2. This strength model describes the rate-dependent inelastic behavior of solids (elastoplastic hardening under large strains) within certain ranges of equivalent plastic strain rates and temperatures.

Modelling and predicting high-velocity impact results require a comprehensive approach that uses classical mechanics along with the conservation laws, combined with failure analysis. The mechanisms of material pitting from cavitation bubble collapse as well as material behaviour during cavitation erosion have been successfully modelled [5]. Today’s level of technology allows for direct observation of damage created by a collapse of a single cavitation bubble with high-speed cameras and 3D microscopes [4]. From the recordings at the very fastest acquisition rate, it is determined that the material deforms and then partially relaxes, while a significant deformation remains. Thde whole process is only 2–3 μs long [4,6]. 

The jet velocity range presented in the paper was 100–500 m/s, based on the velocity range presented in the existing literature [9,10,11,12,13]. The presented calculations assume a perpendicular direction of micro-jet impact. The impact at an angle of 90 degrees transfers most of the energy to the material [19]. Due to solver limitations, the temperature change during impact was not accounted. The finite element erosion mechanism was enabled in calculation. The geometric strain limit factor was assumed to be 1.5.

**Figure 3:**
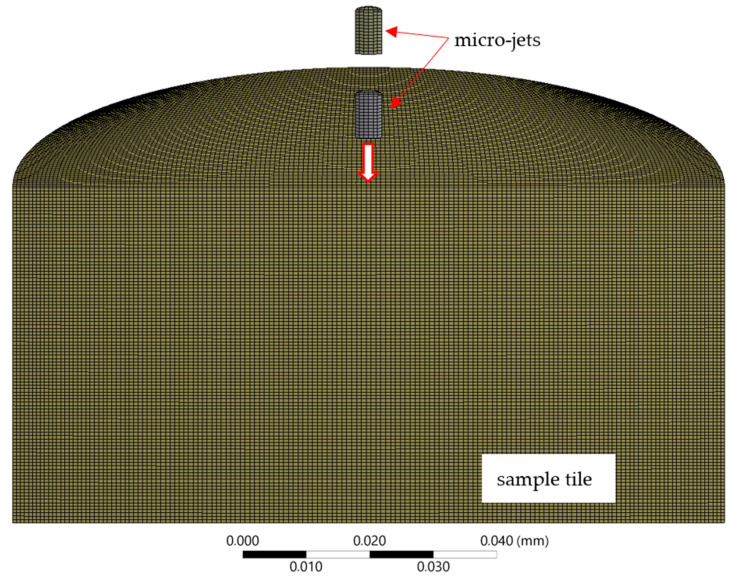
Cross section of numerical calculation domain with FE mesh.

An explicit dynamics simulation code (ANSYS 2020R1) was employed. The tile-shaped sample model was restricted to 40 μm in thickness and 80 μm in diameter (Figure 3). The impact side of the sample was defined as free surface; the two remaining sample surfaces were defined as energy-absorbent impedance boundaries. To reassure initial conditions’ symmetry, the cartesian mesh method was used; the smallest element size accepted by solver was 0.6 μm—measured on elements’ edge. 

The explicit dynamics solver in ANSYS 2020R1 does not accept cyclic nor planar symmetry for the selected mesh type. Therefore, in total, the model had ~1 × 10^6^ hexahedral elements (Figure 3). The size of the micro-jet was based on the results of the experimental observation [17]; the diameter of the micro-jet is often less than one-tenth of the diameter of a cavitation bubble, and the length of the micro-jet is often less than twice the diameter of micro-jet. 

Numerical simulations of the impact velocity and impact pressure derive from the theory of the water hammer [4,20,21]. The impact pressure is determined by two basic equations. Initially, at the moment of impact, the pressure is taken as the water hammer pressure and calculated from the formula

(1) P=ϱcv,

where *ρ* is the density of water; *c* is the acoustic velocity; *v* is the impact velocity. After reaching equilibrium of the incompressible flow in the jet line, the pressure on the center axis drops to a lower level calculated from

(2) $$P=\frac{\varrho v^{2}}{2},$$

where *ρ* is water density. Due to uncertainty in the transition process between initial and equilibrium states, different impact pressure values can be found in the literature. According to Reference [13], the impact pressure reaches about 1 GPa. However, in Reference [5], the maximum impact pressure determined was found to be 3–4 GPa. The duration of an impact depends on the exposed material and varies from 2–3 μs [6] to even 17 μs [7], but a rise time is from 1 μs to 3 μs [13]. Because of the short duration of the impacts, they are called cavitation pulses [8]. Multiple, subsequent impacts of these cavitation pulses on the solid surface cause its damage, leading to material degradation. In the next section, numerical simulation results were compared to available experimental data in order to asses the real-life values of impact velocities and impact pressures.

## 3. Analysis Results

In this section, the measurements of the surface roughness Ra parameter that corresponds to micro-jet pit damage (those results were described in [17]) were compared to the numerical calculation results. The Ra is defined as the arithmetic average of the absolute values of the profile height deviations from the mean line, recorded within the evaluation length. The material specimen’s surface was polished prior to testing Ra = 0.03 µm. In addition, the stress distributions at different stages of micro-jet impact were analysed.

**Figure 4:**
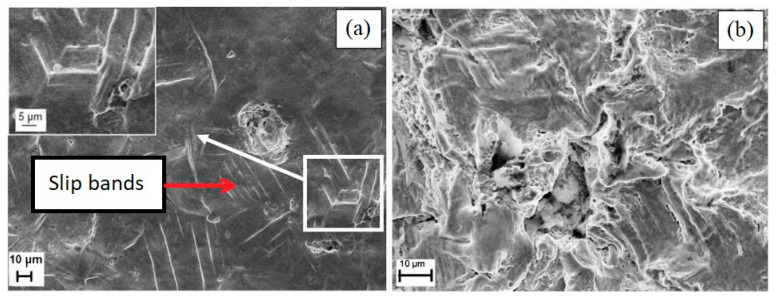
Degradation of 316L steel at low cavitation intensity (p1= 600 kPa): (**a**) Damage in the slightly eroded area; (**b**) Damage in the severely eroded area [17] (Reprinted from “Effect of cavitation intensity on degradation of X6CrNiTi18-10 stainless steel” by A.K. Krella, A. Krupa, Wear, Volumes 408–409, 2018, Pages 180-189, ISSN 0043-1648, Copyright under license no. 5057500490519 (2021), with permission from Elsevier).

According to [17], the cavitation tests were carried out for the inlet pressures p1 = 600, 700, 800, and 900 kPa. The differences in inlet pressures were proportional to the cavitation erosion intensity. The exemplary results of microscopic observations on the specimen’s surface, conducted after 600 min of 600 kPa exposure, are presented in Figure 4. Irregular surface exposition during cavitation test results in differences in surface degradation. Damage in the lightly eroded area is presented in Figure 4a, while damage in the severely eroded is presented in Figure 4b.

The slightly eroded area (Figure 4a) shows slip bands with crevices at intrusions (in the magnified part of Figure 4a), where separate micro-jet impact pits can be observed on the surface of the steel exposed to cavitation at 600 kPa [17]. The diameters of the depressions shown are approximately 5 µm and 50 µm. However, the larger one is likely due to a few micro-jet impacts. This confirms the correctness of the presented approach to the analysis of two successive micro-jet impacts.

In the severely eroded area of the specimen exposed to the cavitation test at p1 = 600 kPa (Figure 4b), a deep void in the mean of damage probably was initiated underneath the surface. Such voids underneath the surface are typically formed due to high shear stresses caused by high-rate impacts [17,22]. This observation indicates the need to determine the stress distribution, in particular under the impact site.

**Figure 5:**
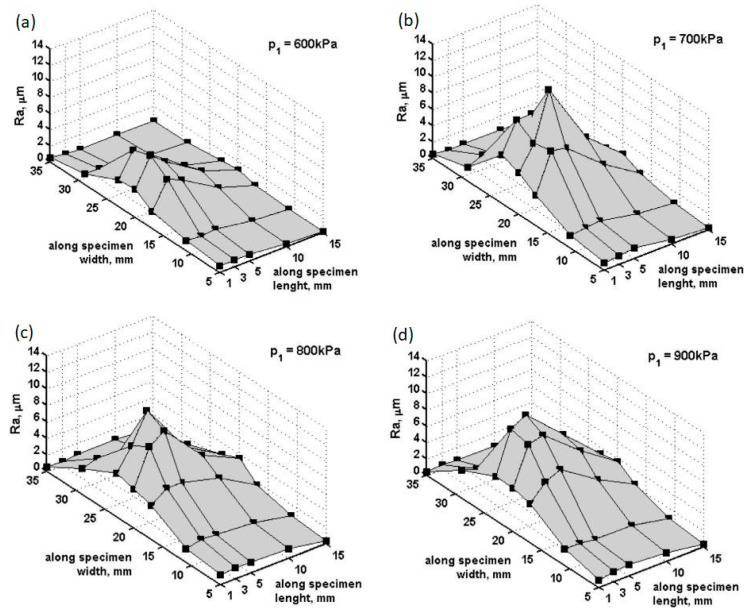
Profile of Ra roughness parameter of 316L steel exposed to cavitation at: (**a**) p1 = 600 kPa; (**b**) p1 = 700 kPa; (**c**) p1 = 800 kPa; (**d**) p1 = 900 kPa (marked points show the arithmetic mean calculated from the values of experimental data) [17] (Reprinted from “Effect of cavitation intensity on degradation of X6CrNiTi18-10 stainless steel” by A.K. Krella, A. Krupa, Wear, Volumes 408–409, 2018, Pages 180-189, ISSN 0043-1648, Copyright under license no. 5057500490519 (2021), with permission from Elsevier).

The arithmetic mean of surface roughness measurement values (micro-jet impact pit depths) were calculated from the experimental data (Figure 5). The inlet pressure/the flow velocity have an influence on the location and the value of surface roughness (Ra parameter). The lowest roughness was present on the specimen exposed to the cavitation test at the lowest inlet pressure, p1 = 600 kPa, which corresponds to flow velocity of 23.04 m/s. The maximum surface roughness of 316L steel after the test was Ra = 7.03 µm, which was detected at approximately 3 mm from the water inlet and approximately 20 mm from the specimen edge (Figure 5a). Taking into consideration the surface roughness profile (Figure 5a) and erosion damage (Figure 4a), low surface roughness was an effect of the shallow micro-jet impact pits [17]. 

An increase in the inlet pressure to 700 kPa, corresponding to a flow velocity of 24.92 m/s, caused an equivalent increase in surface roughness to the value of Ra = 13.98 µm and moved its location from 3 to 5 mm from the water inlet (Figure 5b). Consequently, this resulted in an increase in the inlet pressure from 600 kPa to 700 kPa, nearly doubling the maximum surface roughness. However, the zone of such increased roughness was small. In the distance to 10 mm from the water inlet, the surface roughness reduced to Ra = 6.3 µm. The changed location of the high surface roughness zone together with an increase in maximum surface roughness designate the development of the cavitation erosion phenomenon. A further increase in the inlet pressure did not change the location of the maximum surface roughness, but caused its reduction (Figure 5c,d). The maximum observed surface roughness was Ra = 11.5 µm for both inlet pressures p1 = 800 (flow velocity 26.67 m/s) and 900 kPa (flow velocity 28.29 m/s). Here, according to increasing inlet pressure/flow velocity, the entire exposed surface area further increased; however, the maximum surface roughness was reduced, respectively (Figure 5c,d). For p1 = 900 kPa, almost the entire specimen surface suffered erosion. A decrease in the maximum surface roughness was likely caused by overlapping damage [17]. 

The relation between load intensity and material degradation is still undetermined. Parameters such as the location of bubble collapse, its initial size, and the local collapse driving pressure gradient correspond to the resulting pit geometry (all of these parameters influence the impact load). Load intensity depends on the number and power of cavitation micro-jet pulses [3,9,14,16]. In flow cavitation, the number and properties of cavitation bubbles depend mainly on flow velocity. An increase in the flow velocity increases the number and amplitude of cavitation pulses, gradually leading to higher material degradation [23]. It can be assumed that, with an increase in the flow velocity, the micro-jet impact speed increases. Measurements of surface hardness and damage observations over time indicated the need to analyse the stresses’ development in 316L steel resulting from micro-jet impacts. Taking into account cavitation erosion phenomena, such conditions were met by the two subsequent collisions of high-velocity micro-jets. 

The dynamic explicit FE method was used to estimate load parameters from cavitation pit parameters, taking into account strain rate effects. The correspondence between calculated and generated-in-experiment impact loads was discussed in Section 3.1, Section 3.2 and Section 3.3.

Damage caused by three different impact velocities, 100, 200, and 500 m/s, were chosen as a representation of the effects of micro-jet impact erosion. The calculated von Mises equivalent stress distributions developed in 316L stainless steel during two subsequent micro-jet impacts were presented in three snapshots (stages): at the moment of contacting the surface (first stage), in the middle of impact duration (second stage), and the state after the impact (third stage). As mentioned, a permanent deformation or pit is formed when the local equivalent stresses exceed the material yield stress (here: 307 MPa), resulting in the dislocation movements, initiation of slip, twinning or phase transformations, and growth cracks in metals. The plastic deformation occurs (micro-yielding); atomic planes slip past each other through the movement of dislocations. The direct numerical simulation of these features is currently beyond the capabilities of any FE solver; however, a properly selected set of material data (expressing the spatial averaged values of the mentioned features) and simulation strategy (calculating the amount of energy released by micro-jet impact) allows for reasonable representation of cavitation erosion effects. A comparison between simulated and experimental impact effects allows for the estimation of the micro-jet impact velocities.

### 3.1. Micro-Jet Impact at 100 m/s

**Figure 6:**
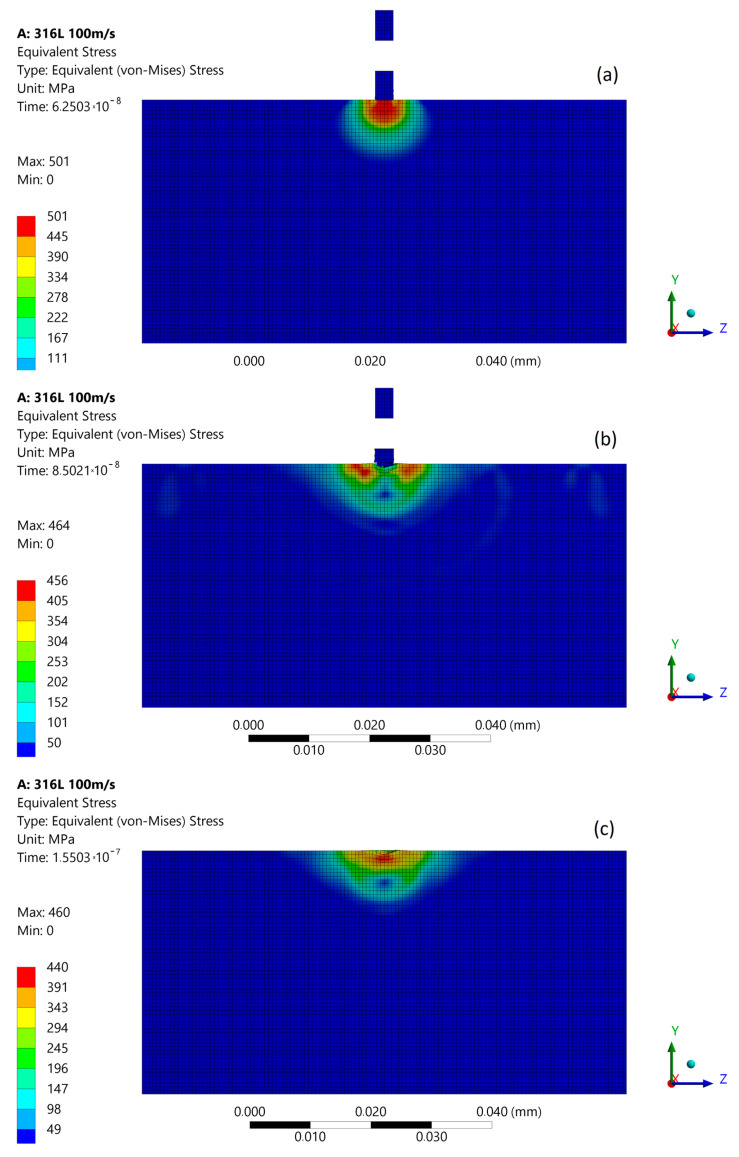
Von Mises stress distribution during first micro-jet impact at 100 m/s: (**a**) initial stage of the impact; (**b**) stage during the impact; (**c**) residual stress after the impact.

The numerical calculation of the micro-jet impact at 100 m/s creates the maximum stress of 501 MPa that develops in the first stage (Figure 6a). The elastoplastic shockwave stresses resulting from the impact began to develop. In the second stage of impact, the maximum stress was reduced to 464 MPa (Figure 6b). The stress distribution was almost uniform as in the first stage, and a local zone of lowered stresses occurs. It can be expected that after impact, the stress levels in the material will decrease in time, according to the Equations (1) and (2) predictions. After the impact (Figure 6c), the maximum residual stress was 460 MPa, and the zone affected by enlarged stress was approximately 9 µm in diameter. As a result of this impact, a 0.7 µm deep pit was formed.

**Figure 7:**
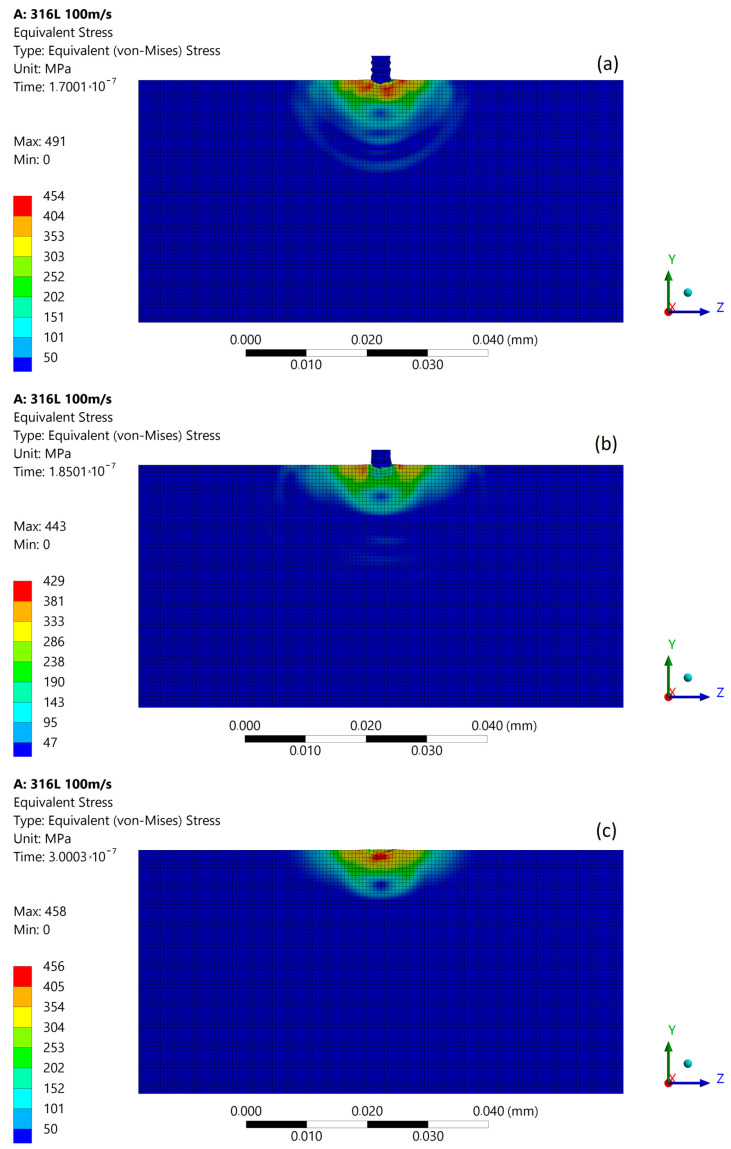
Von Mises stress distribution during subsequent micro-jet impact at 100 m/s: (**a**) initial stage of the impact; (**b**) stage during the impact; (**c**) residual stress after the impact.

The subsequent micro-jet impact at 100 m/s creates the maximum stress of 491 MPa that develops in the first stage (Figure 7a). In the second stage of impact, the maximum stress was reduced to 443 MPa (Figure 7b). The stress distribution was not uniform as in the first stage, and a local zone of low stresses occurs. The stress levels in the material decreased in time, as before. After the impact (Figure 7c), the maximum residual stress was 458 MPa and the zone of enlarged stress remained approximately 9 µm. The lowered level of residual stress after the subsequent impact can be explained by Hertzian contact theory [24], as the contact between second micro-jet and surface of the sample changed to a spherical type. As a result of the subsequent impact, a 0.7 µm depth of pit remained unchanged.

**Figure 8:**
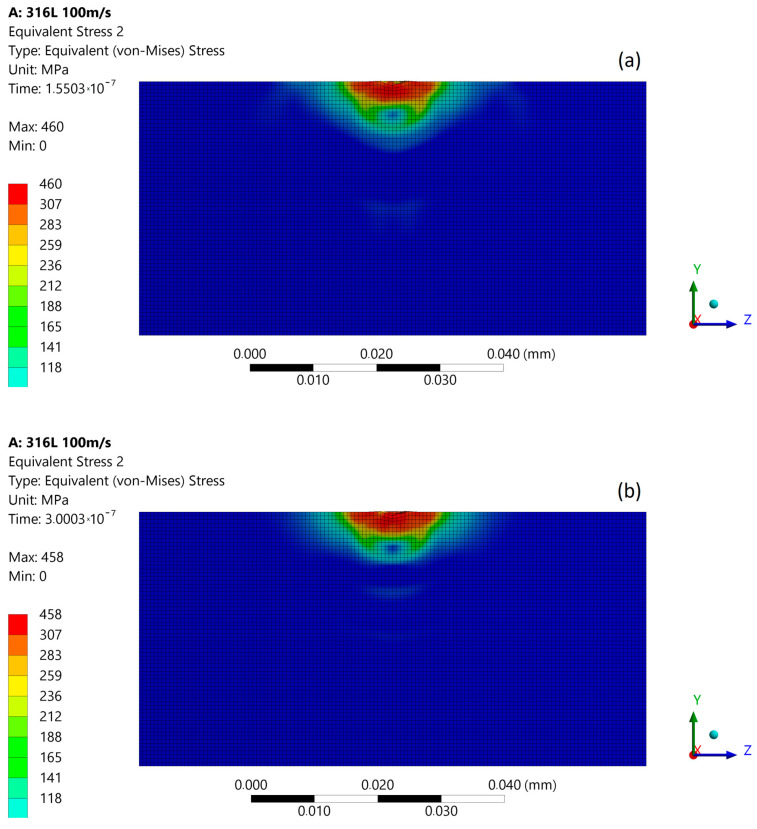
Exceeded yield stress zone (over 307 MPa—red) of 316L stainless steel: (**a**) after first micro-jet impact; (**b**) after subsequent micro-jet impact; at 100 m/s.

The simulation results obtained for the micro-jet impact at 100 m/s are most closely related with experimental results after 600 kPa exposition located in the slightly eroded area (Figure 5a). Figure 8 presents the size comparison between zones of exceeded yield stress (red) created after the first and subsequent micro-jet impact. Here, the yield stress zones were comparable (3.6 µm deep); thus, the elastoplastic hardening effect due to the subsequent micro-jet impact was sufficient to prevent the material from further deformation (strain hardening) [2].

### 3.2. Micro-Jet Impact at 200 m/s

**Figure 9:**
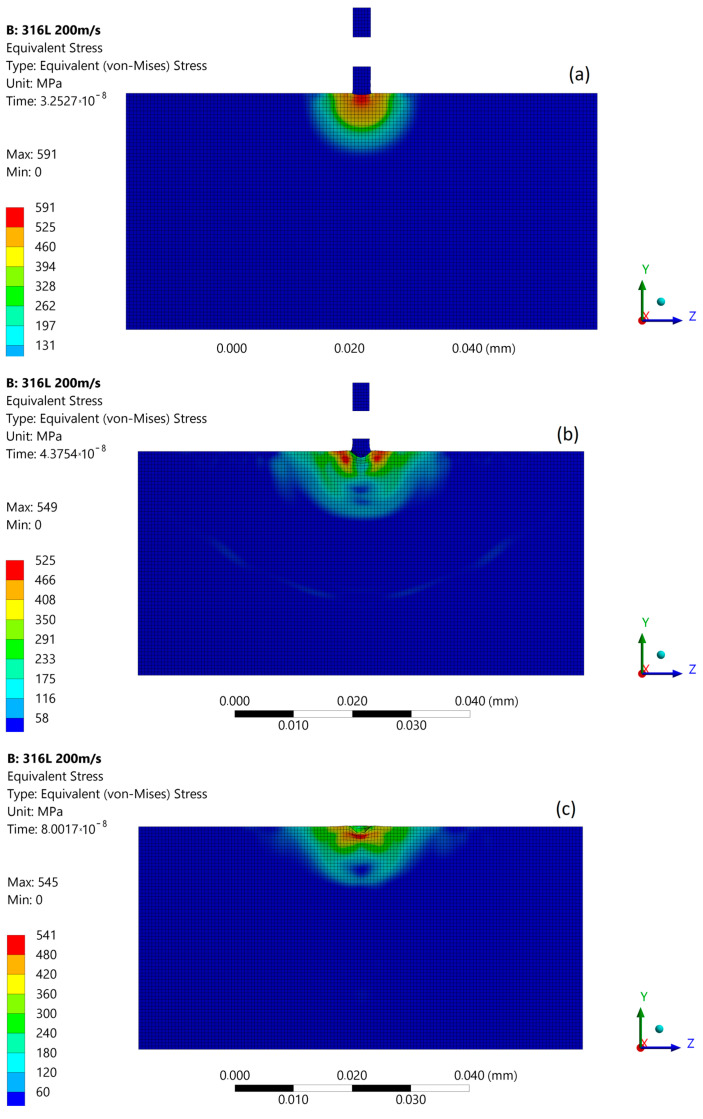
Von Mises stress distribution during first micro-jet impact at 200 m/s: (**a**) initial stage of the impact; (**b**) stage during the impact; (**c**) residual stress after the impact.

With increased impact velocity to 200 m/s, the maximum stresses at the moment of the first micro-jet impact increase to 591 MPa, and the depth of pit amplifies to about 1.2 μm (Figure 9a). In the second stage of impact, the maximum stress was reduced to 549 MPa (Figure 9b). The stress distribution was almost uniform as in the first stage, and a local zone of lowered stresses occurs. After the impact (Figure 9c), the maximum residual stress was 545 MPa, and the zone affected by enlarged stress was approximately 10 µm in diameter.

**Figure 10:**
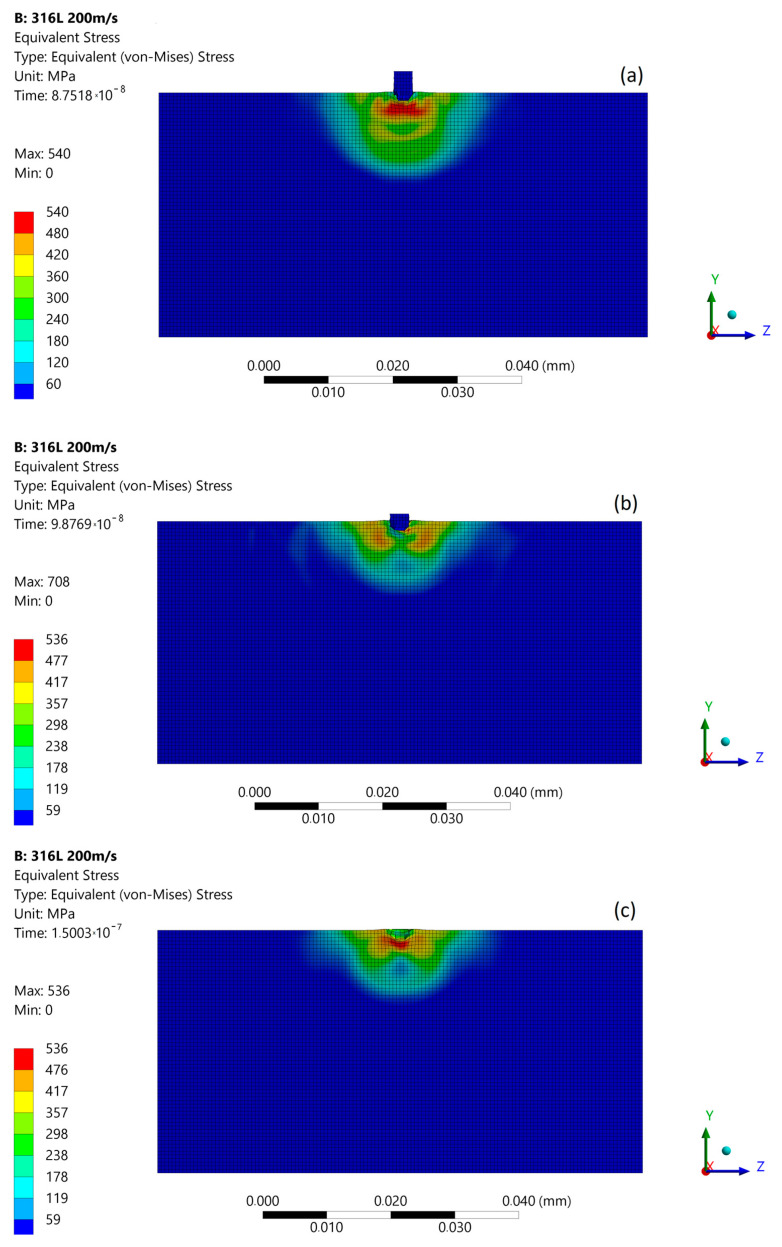
Von Mises stress distribution during subsequent micro-jet impact at 200 m/s: (**a**) initial stage of the impact; (**b**) stage during the impact; (**c**) residual stress after the impact.

**Figure 11:**
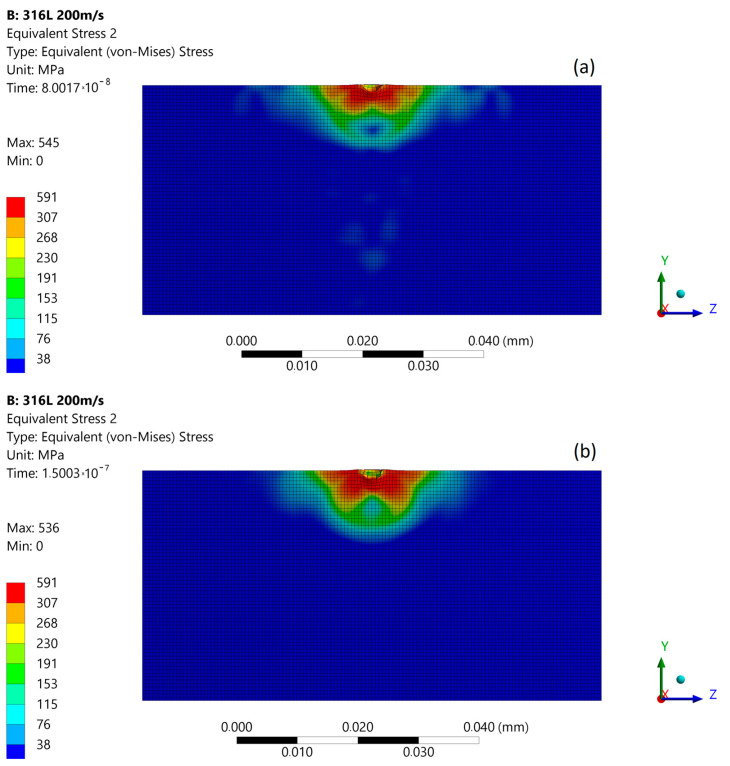
Exceeded yield stress zone (over 307 MPa—red) of 316L stainless steel: (**a**) after first micro-jet impact; (**b**) after subsequent micro-jet impact; at 200 m/s.

The subsequent micro-jet impact at 200 m/s creates the maximum stress of 540 MPa that develops in the first stage (Figure 10a). In the second stage of impact, the maximum stress was momentarily increased to 708 MPa (Figure 10b). The stress distribution was not uniform as in the first stage, and a local zone of high stresses occurs. After the impact (Figure 10c), the maximum residual stress was 538 MPa, and the zone of enlarged stress was approximately 11 µm. As a result of the subsequent impact, a 1.3 µm depth of pit remained. Figure 11 presents the size comparison between zones of exceeded yield stress (red) created after first and subsequent micro-jet impact. In the case of 200 m/s impact velocity, the exceeded yield stress zone was enlarged after subsequent impact. Therefore, the elastoplastic strain hardening was insufficient to prevent the material from further deformation.

The simulation results obtained for the micro-jet impact at 200 m/s are related with experimental results for over 700 kPa expositions located between slightly and highly eroded areas (Figure 5b–d). 

### 3.3. Micro-Jet Impact at 500 m/s

**Figure 12:**
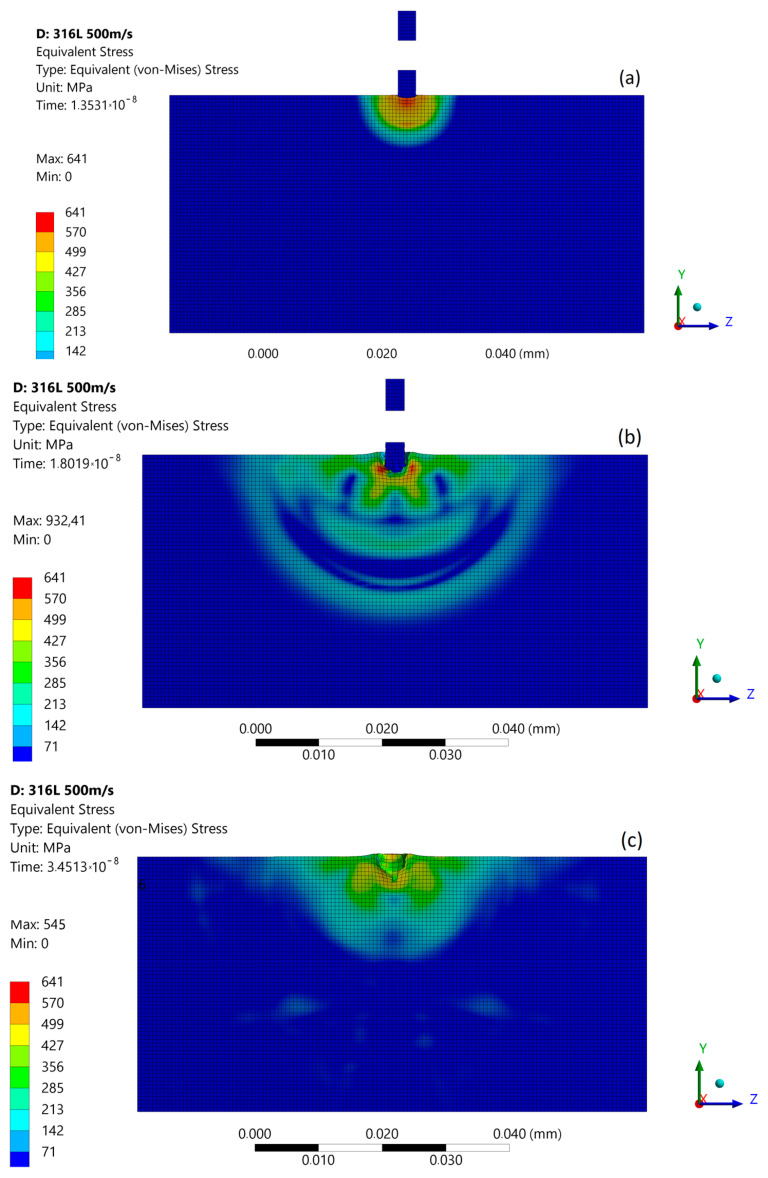
Von Mises stress distribution during first micro-jet impact at 500 m/s: (**a**) initial stage of the impact; (**b**) stage during the impact; (**c**) residual stress after the impact.

With an increased impact velocity to 500 m/s, the maximum stresses at the moment of the first micro-jet impact increases to 641 MPa, and the depth of pit amplifies to about 1.2 μm (Figure 12a). In the second stage of impact, the maximum stress reaches to 932 MPa, triggering the FE degradation process and asymmetric stress distribution (Figure 12b). The impact shock wave was also visible. The estimated stress amplitude of the shockwaves at a 500 m/s micro-jet impact was 250 MPa. After the impact (Figure 12c), the maximum residual stress was 545 MPa, and the zone affected by enlarged stress was approximately 16 µm in diameter. As a result of the subsequent impact, a 3.6 µm depth of pit was created.

**Figure 13:**
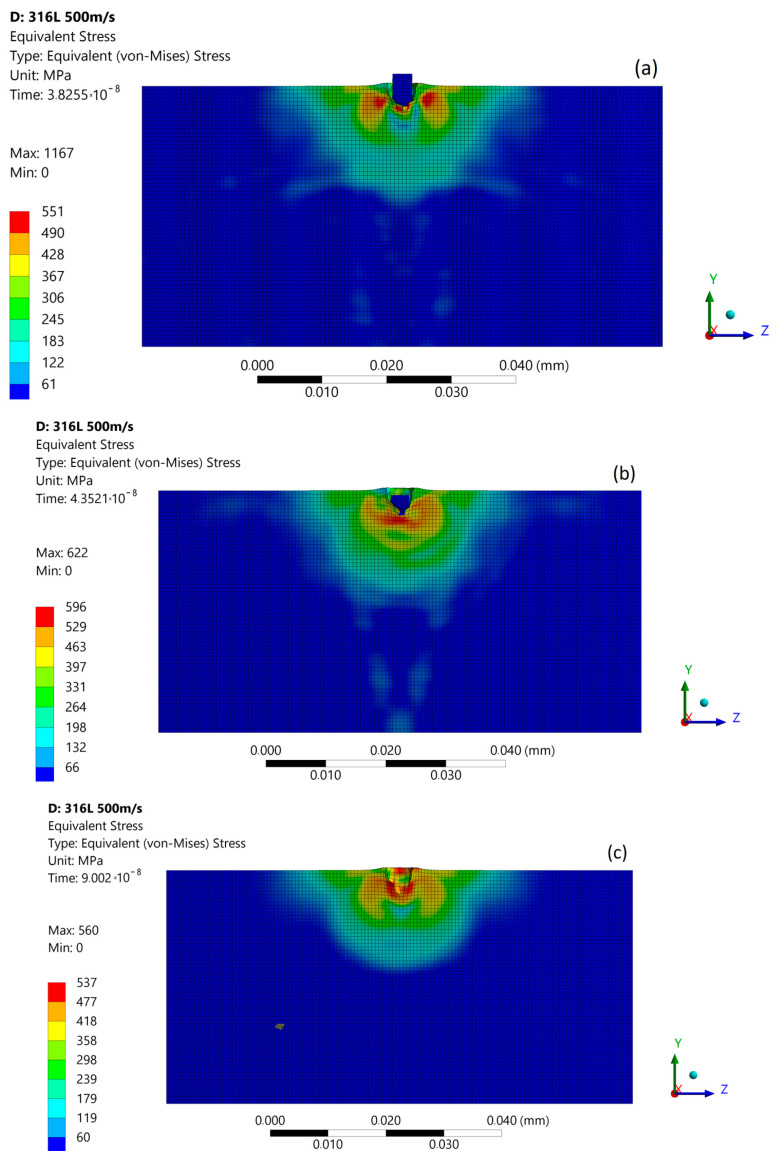
Von Mises stress distribution during subsequent micro-jet impact at 200 m/s: (**a**) initial stage of the impact; (**b**) stage during the impact; (**c**) residual stress after the impact.

**Figure 14:**
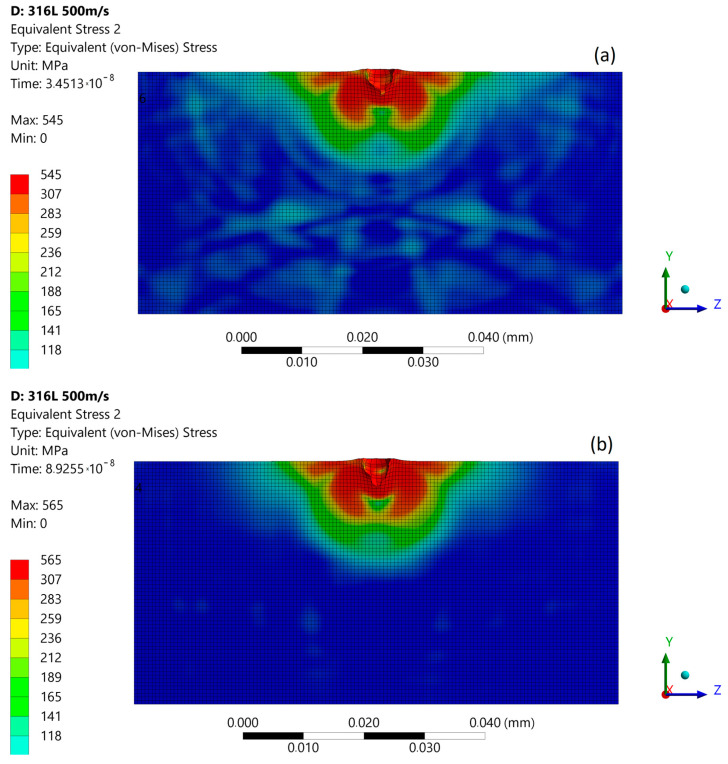
Exceeded yield stress zone (over 307 MPa—red) of 316L stainless steel: (**a**) after first micro-jet impact; (**b**) after subsequent micro-jet impact; at 500 m/s.

The subsequent micro-jet impact at 500 m/s in the first stage creates an impulse of localized stress—1167 MPa—causing FE further degradation (Figure 13a). A high level of material stress acting in austenitic steel is able to initiate a phase change [25]. In the second stage of impact, the maximum stress was decreased to 622 MPa (Figure 13b). The stress distribution was asymmetric as in the first stage. After the impact (Figure 13c), the maximum residual stress was 560 MPa, and the zone of enlarged stress was approximately 18 µm. As a result of the subsequent impact, a 4.5 µm depth of pit remained. Figure 14 presents the size comparison between zones of exceeded yield stress (red) created after the first and subsequent micro-jet impact. In the case of a 500 m/s impact velocity, the exceeded yield stress zone was significantly larger after the subsequent impact. The elastoplastic hardening effect and the exceeded geometric strain limit (which was assumed to be 1.5) caused localised material degradation. The surface softening effect was likely influenced by traveling shock wave. The simulation results obtained for the micro-jet impact at 500 m/s are related with experimental results for over 700 kPa expositions located in highly eroded areas (Figure 5b–d). 

## 4. Discussion

The numerical simulation of the micro-jet impact on complex microscale structures of the materials is currently beyond the capabilities of FE solvers. However, with appropriate simplifying assumptions and with experimental results for comparison, certain conclusions on the material damage characteristics can be derived. The first assumption is that the average micro-jet diameter is not correlated with micro-jet impact speed. This allows the evaluation and comparison of results in reference pit depths. The second assumption is that the material data are expressing the spatial averaged values of the complex microscale structures. This assumption excludes the capability to picture micro-erosion mechanisms (as there is no grain structure) with high accuracy, although it represents the micro-jet impact magnitude correctly (as the deformation is proportional to transferred energy). The resulting stress distribution maps as spatial mean values must be considered as an idealized representation. However, they should give insight into dynamic material behaviour under micro-jet impact.

Performing the calculations of a micro-jet water hammer for impact velocities between 100 and 500 m/s revealed that the depth of the pit varies between 0.7 μm and 3.6 μm, respectively. The erosion pit diameter was formed between 4.2 μm and 4.6 μm, respectively. In the case of the subsequent impact with the velocity of 500 m/s, the depth of a pit increases by 0.9 μm, while pit diameter did not change measurably. The mechanism of pit creation occurs through material deformation or degradation and depends on equivalent stress levels and the magnitude of the elastoplastic hardening effect. The origin of the “elastoplastic” hardening is the strain hardening due to plastic deformation. The material degradation (erosion) formed by a micro-jet water hammer occurs for velocities of 500 m/s and higher, or subsequent impacts at 200 m/s. In simulation, the degradation occurs when the material strain limit is exceeded. In the experiment, the material degradation mechanism is much more complicated. A high level of material stress acting in austenitic steel is able to initiate a phase change. The phase change γ → α’ in metastable austenitic stainless steel can occur due to repeated loading. Due to a low content of carbon in such steel, α’ is formed instead of ε. The brittle fracture originating in high stress concentration regions, even in severely eroded zones, is very unlikely for 316L steel, as shown in Figure 4b; even a high erosion rate causes typical plastic surface deformation.

The impact pit depth comparison between numerical calculation results and 3D profiles of test specimens revealed that the velocity of the micro-jet water hammer in a highly eroded area was 500 m/s. An explanation of the influence of traveling shockwaves, observed after the micro-impact, on the residual stress and/or stress relaxation requires more complex simulations of multiple, subsequent impacts over a larger area and will be the subject of future studies.

## 5. Conclusions

The simulation of two subsequent micro-jet impacts at a 90 degree angle on the 316L stainless steel was performed for impact velocities between 100 and 500 m/s. The resulting, post-impact pit geometry was compared to the experimental data to estimate the micro-jet impact velocity. The relationships between changes in surface deformation, stress distribution, and material hardness were described.

Numerical simulation showed that the depth of the erosion pit formed with the first micro-jet impact was proportional to the impact velocity. At the end of the first impact, the pit depth fluctuated between 0.7 μm and 3.60 μm, depending on impact speed. At the end of the subsequent impact, the pit depth change was between 0.7 mm and 4.50 mm, accordingly. The contact stress generated by the micro-jet with a velocity of 100 m/s was the largest at the moment of contact. Then, stress decreases in the subsequent impact process. The impacts at higher velocities, 200 and 500 m/s, due to asymmetry can cause a momentary stress increase and FE degradation leading to further asymmetries. 

Numerical simulation showed that the impact of the water micro-jet with the velocity of 100 m/s does not exceed the tensile strength of 316L stainless steel, and a short-lasting impact produces an indentation (deformation) pit with a depth of 0.7 μm. The micro-jet impact at 100 m/s is most closely related with experimental results after 600 kPa exposition located in a slightly eroded area. 

In the case of 200 m/s impact velocity, the exceeded yield stress zone was enlarged after subsequent impact. Therefore, the elastoplastic hardening was insufficient to prevent material from further deformation. The simulation results obtained for the micro-jet impact at 200 m/s are related with experimental results for over 700 kPa expositions located between slightly and highly eroded areas. 

In the case of an impact with the velocity of 500 m/s, a (degradation) pit with a depth of 3.6 μm was observed. The subsequent impact of a micro-jet water hammer with the same impact velocity increases the depth of the pit to 4.5 μm. Here, the exceeded yield stress zone after the subsequent impact was significantly larger. Therefore, the elastoplastic hardening was insufficient to prevent material from further degradation. The simulation results obtained for the micro-jet impact at 500 m/s are related with experimental results for over 700 kPa expositions located in highly eroded areas. The surface softening effect was likely influenced by the observed traveling shockwave. This phenomenon will be verified in the future studies.

## Figures and Tables

**Table 1:** Chemical composition (%) of steel 316L, according to certificate.

C	Mn	Si	Ni	Ti	Cr	P	S
0.01	1.79	0.53	9.56	0.15	17.05	0.025	0.027

**Table 2:** The material properties of 316L steel used in numerical calculations.

Property	Value/Type	Unit
Density	7990	kg/m^3^
Young’s Modulus	199	GPa
Poisson’s Ratio	0.3	
Bulk Modulus	160.8	GPa
Shear Modulus	74.2	GPa
Specific Heat	452	J/kgK
Strain Rate Correction	First-Order	-
Initial Yield Stress	307	MPa
Hardening Exponent	0.36	MPa
Hardening Constant	275	-
Strain Rate Constant	0.022	-
Thermal Softening Exponent	1	-
Melting Temperature	1537.9	C
Refresh Strain Rate	1	1/s
Bulk Modulus	164	GPa
Shear Modulus	80	GPa
Tensile Strength	662	MPa

## Data Availability

The data presented in this study are available on request from the corresponding author.
